# EPDM Rubber Modified by Nitrogen Plasma Immersion Ion Implantation

**DOI:** 10.3390/ma11050657

**Published:** 2018-04-24

**Authors:** Alexey Kondyurin

**Affiliations:** School of Physics, University of Sydney, Sydney 2006, Australia; aleksey.kondyurin@sydney.edu.au; Tel.: +61-29351-2484

**Keywords:** ethylene-propylene diene monomer rubber EPDM, plasma immersion ion implantation, adhesion, FTIR ATR, interface, polyurethane

## Abstract

Ethylene-propylene diene monomer rubber (EPDM) was treated by plasma immersion ion implantation (PIII) with nitrogen ions of 20 keV energy and fluence from 10^13^ to 10^16^ ions/cm^2^. The Fourier-transform infrared attenuated total reflection spectra, atomic force microscopy and optical microscopy showed significant structure changes of the surface. The analysis of an interface of PIII treated EPDM rubber with polyurethane binder showed a cohesive character of the adhesion joint fracture at the presence of solvent and interpreted as covalent bond network formation between the PIII treated rubber and the adhesive.

## 1. Introduction

Ethylene-propylene diene monomer rubber (EPDM) materials have a number of applications in different fields of industry, for example, profiles, hoses, cable seals, roofing membranes, seal for doors and windows in car and airplane, covering in irrigation systems, gaskets for water systems and others [1,2]. With a low cost, high stability in harsh environment and wide temperature range with suitable properties of elasticity and strength gives advantages for EPDM rubbers to be used in specific fields such as cable transit seals for nuclear reactors [3]. 

Good thermo resistant and thermo protection properties of EPDM rubbers are used in solid rocket engine as a thermo-protecting layer between a rocket wall and a propellant [4,5]. Recently, EPDM rubber was considered as a prospective material for heat shielding materials for hypersonic flights in the reentry part of trajectory [6,7]. 

However, high inertness and stability of the EPDM rubber is a disadvantage in low adhesion of the rubber. Most reactive adhesives cannot provide sufficient adhesion strength to the EPDM rubber [8,9,10,11]. Under critical conditions of wide temperature range, the presence of plasticizer and a high mechanical load, a surface modification of EPDM rubber is essential to get the sufficient adhesion [12]. 

One of the well-known and well-developed methods to improve the adhesion of the polyolefin is plasma discharge [13,14]. Plasma methods are used for modification of the polyolefin surface for improvement of wettability, painting, hardness, chemical activity and also to improve adhesion [15,16,17,18,19]. The plasma treatment of EPDM rubber was used to improve hydrophilicity [20] and to decrease friction [21]. The plasma methods were used for improvement of adhesion of EPDM thermal protection coating to polymeric binder of the propellant in solid fuel rocket engines [22,23]. The plasma treatment of EPDM rubber causes oxidation and nitrization of the thin surface layer, improves the surface wettability due to new carbonyl and carboxyl groups on the surface and increases the surface roughness. These structure changes are considered a reason for improved adhesion [24,25,26]. A similar effect where the surface structure changes in EPDM rubber was observed after plasma immersion ion implantation (PIII) [27]. The surface energy of EPDM rubber increases together with roughness of the surface, and oxygen-containing groups appearing on the surface. 

However, the structure of the EPDM after the plasma treatment is changed, due to free radical reactions [28]. The free radicals are much more active with the hydrocarbon macromolecules. The reactions of free radicals to the polymer binder leads to a formation of a covalent bond. This can provide a strong adhesion under critical conditions of wide temperature range, presence of plasticizer, high mechanical load and long storage time [29]. 

Plasma immersion ion implantation is a powerful method of surface modification of polymer material [30]. The ions from a plasma cloud are accelerated towards a polymer surface. The kinetic energy of ions exceeds the chemical bond energy of polymer macromolecule in some orders of magnitude. As a consequence of the PIII, the surface layer is highly carbonized and reacts with oxygen and nitrogen in open air. The thickness of the modified layer corresponds to the penetration depth of ions into the polymer [30]. 

Using this method, the structure of materials in the thin surface layer had been changed dramatically while the bulk structure was left intact. This is important in the various fields of application, when the combination of opposite properties must be provided in one material. One of these properties is adhesion of inert materials like polyethylene, polytetrafluorethylene and other polymers in an aggressive environment. The stable adhesion of polymer materials under mechanical stress requires a strong adhesion interaction based on a chemical mechanism of an interface formation, which provides sufficient adhesion strength in wide temperature range of exploitation as well as in aggressive media including strong swelling agents. At the same time, the modified materials must provide required mechanical strength, elastic properties, thermostability, thermoelasticity, thermodynamical compatibility and sometimes a number of exotic properties like predictable behavior in ablation processes which are related to the volume properties of polymer. 

In the present paper, we investigate the EPDM rubber without any additives, which are usually used to achieve the required technological properties. The goal of this study is to observe an influence of PIII treatment on EPDM rubber surface and interface in an adhesion joint with polyurethane binder. 

## 2. Materials and Methods 

EPDM rubber contained 59% of ethylene chains, 39% of propylene chains and 2% of dicyclopentadiene chains was used for the 20 × 30 mm^2^ plates of 2 mm in thickness. The rubber did not contain plastisizers, additives, vulcanizing agents, fillers and other components. The vulcanization was done by γ-irradiation from ^60^Co source [31,32,33]. 

PIII treatment of EPDM rubber was done with using of nitrogen plasma generated by electron cyclotron resonance source (Rossendorf Research Center, Dresden, Germany). The plasma density measured by Langmuir probe was 10^10^ cm^−3^ and 9 eV electron temperature. The rubber samples were placed on 60 × 100 mm^2^ high voltage electrode and covered with a metal grid electrically connected to the electrode. The grid had 0.3 mm cell dimension and a wire thickness of 0.05 mm. The distance between the grid and rubber surface was 20 mm. This scheme provided an absence of shadow effect of the grid on the rubber surface. The high voltage pulse of 20 kV and duration of 5 μSec was applied. The frequency of pule repetition was selected to avoid the sample overheating. The temperature of the samples treated with the highest fluence of 10^16^ ions/cm^2^ didn’t exceed 30 °C. The ion fluence was calculated from the amount of high voltage pulses multiplied on a fluence of one pulse. The one pulse fluence was calculated by the method of UV spectra absorbance of polyethylene samples with known fluence from ion beam source [30]. 

For a comparison, the PIII treatment of EPDM was done without the grid. In such a case, the EPDM samples were placed on the electrode but not covered by the grid. The treatment was done at the same parameters and time of the PIII treatment. 

FTIR ATR spectra in a range of 400–4000 cm^−1^ was recorded after 30 days in storage in covered boxes to avoid contamination and light illumination. The spectrometer Nicolet Magna 650 was used. ATR spectra were recorded with ATR crystal Ge, the angle of the beam incident was 45 degrees, and number of scans was 100. The spectral resolution was 1 cm^−1^. 

Microphotos of rubber surface were done with using of optical microscope MBS-10 attached video camera.

AFM images were measured by using an atomic force microscope Rasterscope C-21 (DME, Herlev, Denmark) with software Dual Scope/Rasterscope SPM 1.3.2.

## 3. Theory

**Figure 1:**
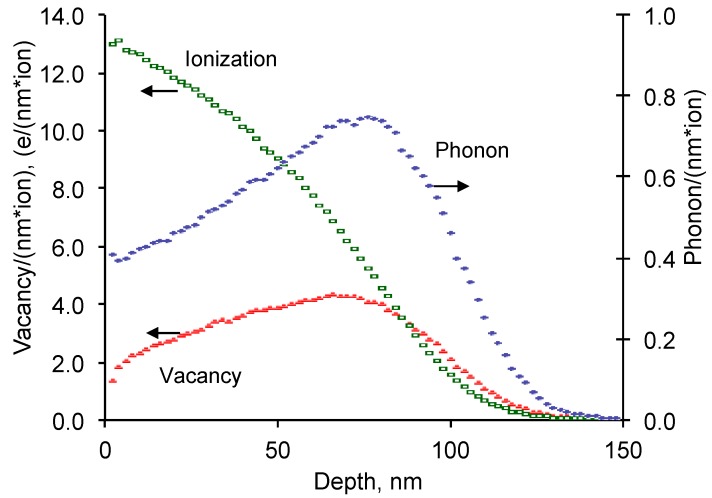
Ionization, phonon and total vacancy profiles after nitrogen ion of 20 keV energy penetrating into EPDM rubber. By SRIM calculations.

The theoretical calculations of ion penetration and atomic collisions in EPDM rubber were done using a SRIM software code [34,35]. The calculations were based on a model of atomic and electronic collisions of the target macromolecule with the penetrating ions. A kinetics energy of the penetrating ion was high enough to recoil the electrons and atoms from the mother macromolecule and to transfer a part of the kinetic energy to the recoiled particles in a such a way, that the recoiled particles can also travel fast enough to collide and to recoil other electrons and atoms in the polymer target. With a number of collisions and energy transfers the ion and recoiled electrons and atoms were stopped. These cascades of collision and energy transfers were observed in a thin surface layer, which the depth depends on the kind and the initial kinetic energy of the penetrating ion and the kind ad density of the polymer target. The distribution of the electron collisions, atom recoiling and phonon energy transfer for EPDM rubber is shown on Figure 1. The electron collisions dominated on the surface and decayed with the penetration depth. The electron collisions mostly caused ionization of the macromolecules that did not cause a structure transformation after it. While the electron with high energy can cause secondary collisions with atoms, the probability of such processes is low in comparison with the atomic collisions under considered conditions.

**Figure 2:**
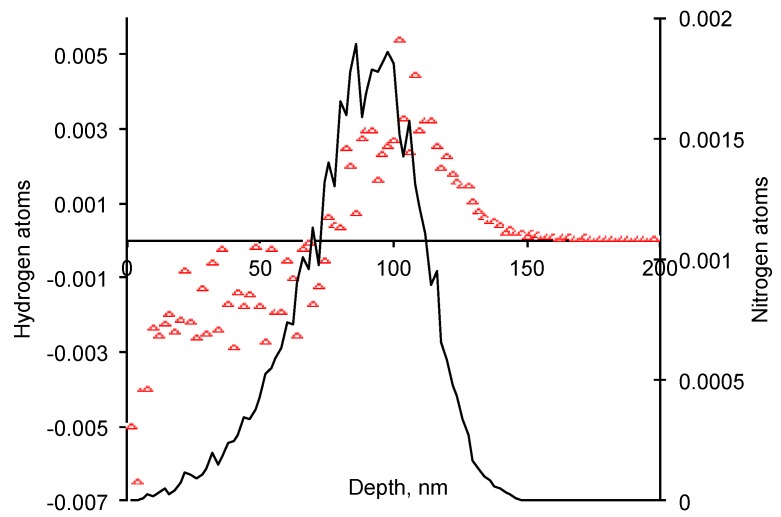
Stopped nitrogen ion profile (black line, units are stopped nitrogen atoms/nm^3^ per penetrated nitrogen ion/nm^2^) and hydrogen atoms balance (units are replaced hydrogen atoms/nm^3^ per penetrated nitrogen ion/nm^2^) with depth of EPDM rubber. By SRIM calculations.

**Figure 3:**
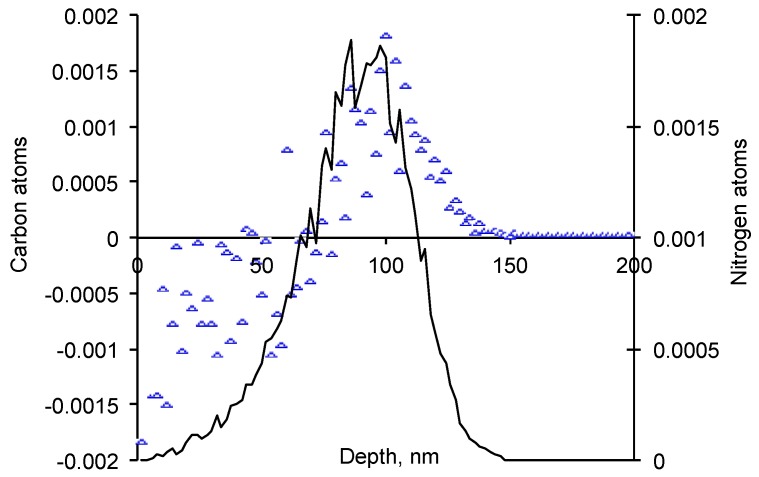
Stopped nitrogen ion profile (black line, units are stopped nitrogen atoms/nm^3^ per penetrated nitrogen ion/nm^2^) and carbon atoms balance (units are replaced carbon atoms/nm^3^ per penetrated nitrogen ion/nm^2^) with depth of EPDM rubber. By SRIM calculations.

The atomic collisions can break the chemical bonds and release the atoms from the mother macromolecule. The recoiled atom gets some kinetic energy and impulse. After a certain distance from the mother macromolecule the recoiled atom stops due to interactions with other macromolecules. The shift of the atom position for hydrogen and carbon atoms in EPDM rubber is presented in Figure 2 and Figure 3. The thin surface layer about 70 nm thick becomes poor with these atoms. Due to recoiling the mother macromolecule gets an unpaired electrons at left atoms called free radicals. Therefore, this surface layer becomes porous and active with free radicals.

**Figure 4:**
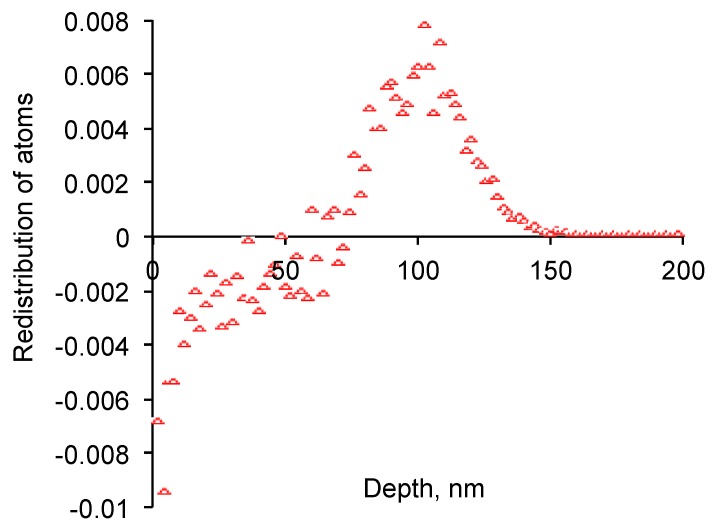
The total atoms balance (nitrogen, carbon and hydrogen, units are replaced atoms/nm^3^ per penetrated nitrogen ion/nm^2^) with depth of EPDM rubber. By SRIM calculations.

The thicker layer between 70 and 130 nm where the recoiled atoms are stopped gets more hydrogen and carbon atoms as well as stopped nitrogen ions (Figure 4). The stopped atoms have also unpaired electrons: carbon has four, nitrogen has three and hydrogen has one. Therefore, this layer becomes denser and active with free radicals. The stopped atoms with unpaired electrons can join to each other and other local macromolecules on the way of the free radical reactions. A structure like aromatic rings with nitrogen inclusions up to graphitic planes or carbon spheres like fullerene structures can be expected there. A migration of free radicals along the EPDM backbone can be expected as well as the effect of the macromolecules crosslinking in a deeper layer than calculated. 

A positive balance of the atomic concentration in this layer can cause tensile force in the surface layer. The stresses in thin surface layer with elastic underneath layer of the rubber can cause a deformation up to folds or waves. The results of the theoretical consideration show that the treated surface layer should have different structure and properties in comparison with the untreated one. 

## 4. Results

The untreated EPDM rubber is light-brown color. After PIII treatment with low fluence, the color does not change. At 10^15^ ions/cm^2^ and higher fluence the color in reflection light becomes silver-like corresponding to the carbonized surface. The same effect of the color change was observed for other polymers after ion beam treatment [30]. 

**Figure 5:**
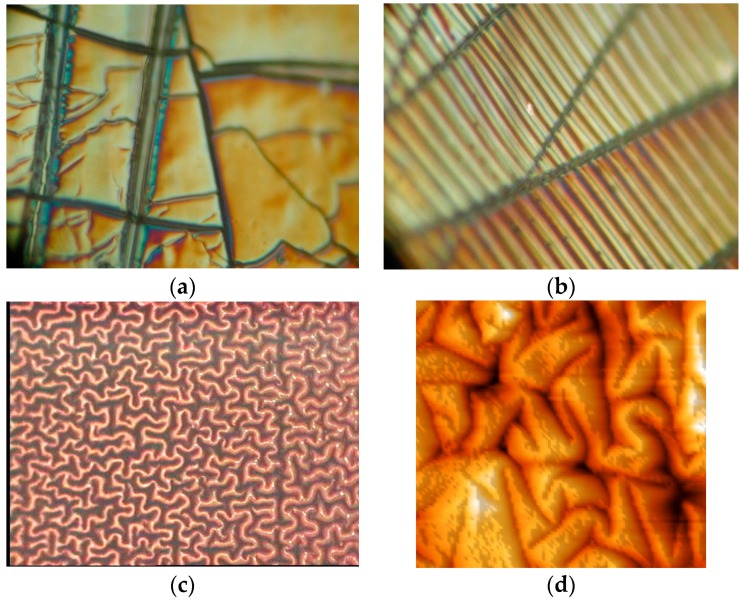
Optical microphotograph (**a**–**c**, 20 × 24 μm) and AFM image (**d**) of EPDM rubber after PIII: (**a**) 10^14^ ions/cm^2^ PIII treated EPDM; (**b**) 10^15^ ions/cm^2^ PIII treated EPDM; (**c**) 10^16^ ions/cm^2^ PIII treated EPDM; (**d**) AFM image of 10^16^ ions/cm^2^ PIII treated EPDM (7 × 7 μm with 244 nm height amplitude).

**Figure A1:**
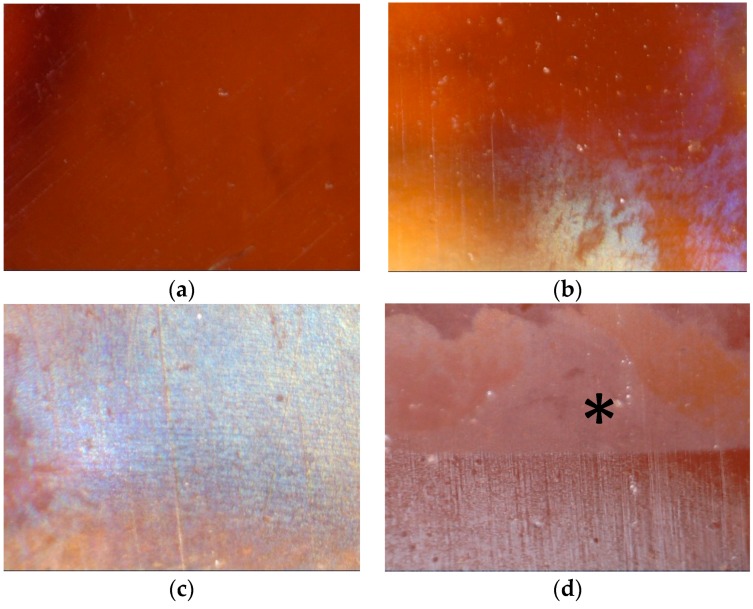
Optical microphotograph (2 × 1.5 mm) of EPDM rubber after PIII: (**a**) untreated; (**b**) 5 × 10^13^ ions/cm^2^ PIII treated EPDM; (**c**,**d**) 10^14^ ions/cm^2^ PIII treated EPDMd (**d**) the edge of the mask (* marks the treated area).

**Figure A2:**
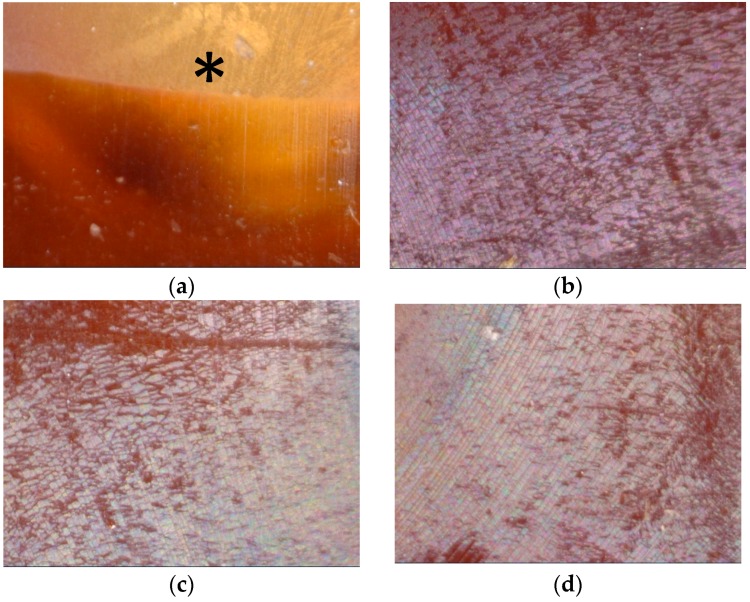
Optical microphotograph (2 × 1.5 mm) of EPDM rubber after 5 × 10^14^ ions/cm^2^ PIII. (**a**) the edge of the mask (* marks the treated area). (**b**–**d**) are in a center of the treated area.

**Figure A3:**
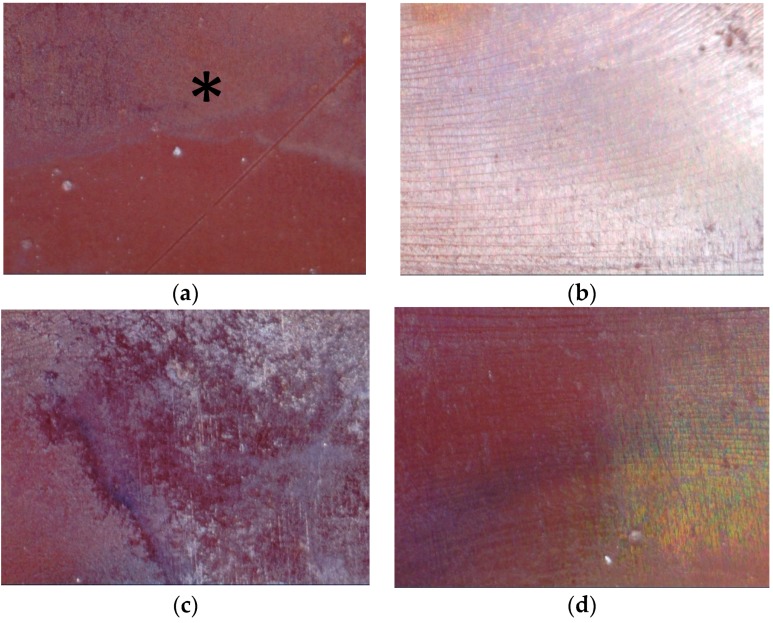
Optical microphotograph (2 × 1.5 mm) of EPDM rubber after 10^15^ ions/cm^2^ PIII, (**a**) the edge of the mask (* marks the treated area). (**b**–**d**) are in a center of the treated area.

**Figure A4:**
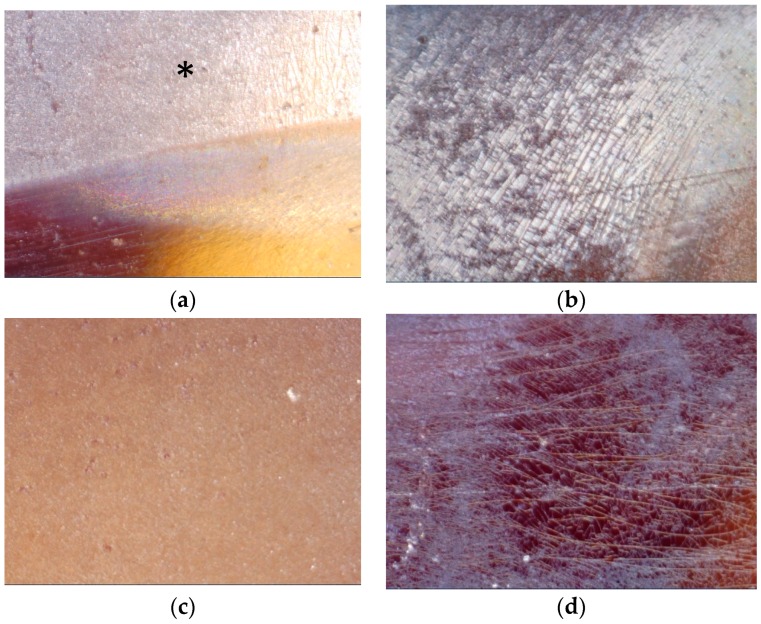
Optical microphotograph (2 × 1.5 mm) of EPDM rubber after 5 × 10^15^ ions/cm^2^ PIII, (**a**) the edge of the mask (* marks the treated area). (**b**–**d**) are in a center of the treated area.

**Figure A5:**
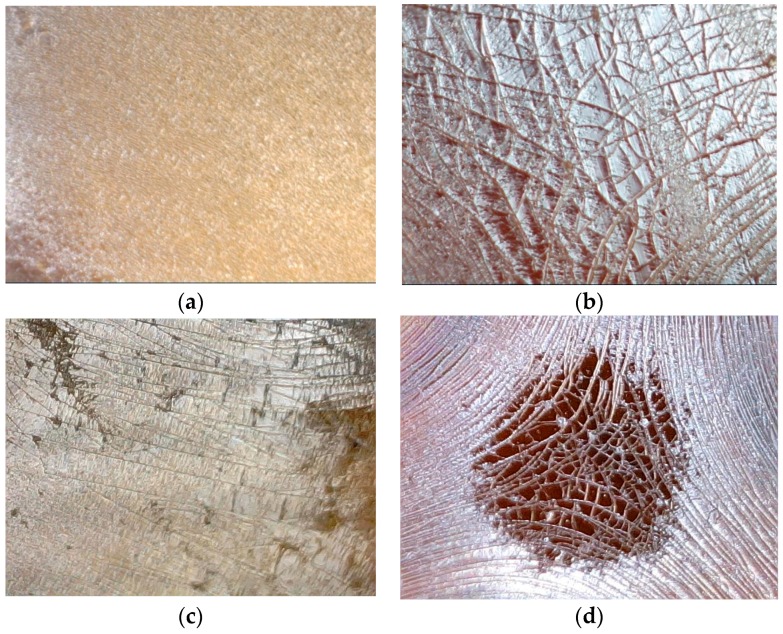
Optical microphotograph (2 × 1.5 mm) of EPDM rubber after 10^15^ ions/cm^2^ PIII. (**a**–**d**) are in a center of the treated area.

Optical microphotos of the EPDM samples show that the surface topography significantly changes after PIII (Figure 5). The surface of untreated EPDM rubber is smooth (Figure A1). After PIII treatment the surface becomes rough with different kinds of structures dependent on the fluence of the treatment (Figure A2, Figure A3, Figure A4 and Figure A5). 

The treated surface becomes to be covered by a net of folds and cracks. The cracks are viewed as a hard thin surface layer on soft bulk substrate was deformed up to breaking. The reason of there kinds of cracks is the carbonization of the surface of the thin layer under ion beam and formation of brittle layer which is jointed to the elastic bulk layer of unchanged rubber. The reason of folds is the stresses in the carbonized layer, which deform the surface layer at presence of elastic underneath layer [36]. Similar surface topography changes were observed for elastic polymers after ion beam treatment [27,37,38]. However, the observed topography of EPDM rubber after PIII is different in comparison with the EPDM surface etched in low voltage plasma treatment [26]. 

At low fluence (5 × 10^13^ ions/cm^2^) the surface is changed minor (Figure A1b). Only some area is observed different than the untreated surface. With fluence growing up to 10^15^ ions/cm^2^ the surface becomes covered by cracks and folds (Figure 5a). The rough fields are distributed irregular on the surface: some area is covered by ditches and hills while some area remains smooth. In microscope image, the folds have silver-like color (Figure A1, Figure A2, Figure A3, Figure A4 and Figure A5) and in some places they are surrounded by areas with colorful areas of interference light (Figure 5a). Other areas of the sample remain light-brown corresponding to the color of unmodified rubber. The width of the folds is about 0.5–5 μm. With fluence increase the folds become deeper and wider and the density of the folds increase. The whole surface becomes to be covered by folds and cracks at fluence higher than 10^15^ ions/cm^2^. 

In some areas the cracks are locally oriented in parallel to each other and the folds are oriented to perpendicular to the cracks forming a ladder structure (Figure 5b). The folds have an elongated form and they are regularly arranged in parallel order to each other. In some areas, the folds form a worm-like structures and completely disordered arrangement (Figure 5c). Such worm-like folds cover about 50% of the rubber surface at 10^16^ ions/cm^2^ high fluence of PIII treatment (Figure A4c and Figure A5a). 

The cracks appear at low fluences and release the contracting stresses crossing the cracks. The folds appear at higher fluence and release the stresses perpendicular to the cracks. In such areas, the distributions of folds depend on the cracks. However, in other areas the worm-like structures show only folds without cracks. Also some areas show only with cracks without folds (Figure A4d and Figure A5d) even at highest fluences. 

Such different surface structures after the PIII and ion beam treatment were not observed in polyethylene or other polymers with homogeneous composition [30]. The difference in the surface structures can be caused by inhomogeneity of initial EPDM composition. Indirect evidence of such reason is a distribution of the different structure near the inclusions of the EPDM samples. However, the non-uniformity of the PIII fluence distribution could play a minor role due to homogeneous surface structures near the edge of metal mask, which was applied to the EPDM samples at the same time of treatment. The border between the untreated area covered by mask and the treated area is sufficiently sharp and does not have a gradient of the structures (Figure A1d and Figure A2a, Figure A3a and Figure A4a). Therefore, the formation of the different local surface structures like cracks and folds depends on the initial EPDM rubber inhomogeneity. 

**Figure 6:**
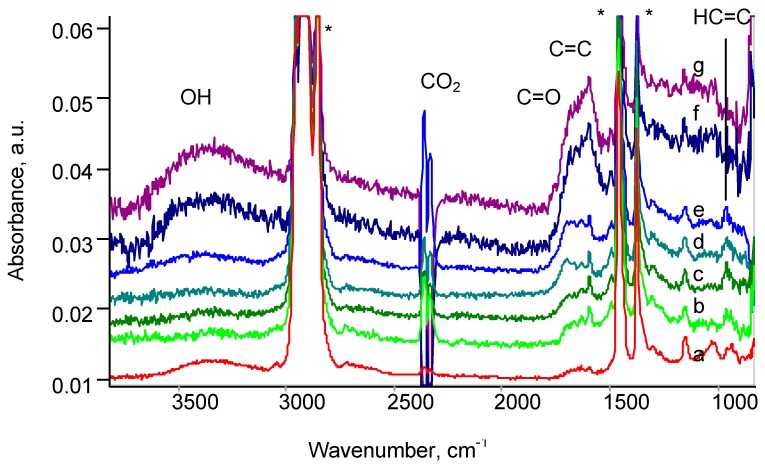
FTIR ATR spectra of EPDM after PIII with different fluence (ions/cm^2^): (**a**) untreated; (**b**) 10^13^; (**c**) 10^14^; (**d**) 5 × 10^14^; (**e**) 10^15^; (**f**) 5 × 10^15^; (**g**) 10^16^. * are the lines of bulk EPDM. “CO_2_” is a line of carbon dioxide in atmosphere of the FTIR spectrometer.

FTIR ATR spectra of untreated EPDM rubber show lines attributed to ethylene and propylene chains vibrations of macromolecules: symmetric and asymmetric stretch ν(C-H) vibration lines at 2923, 2852 cm^−1^ for -CH_2_- and 2950, 2870 cm^−1^ for -CH_3_ groups, deformation δ(C-H) vibrations in -CH_2_- and -CH_3_ groups at 1462 and 1375 cm^−1^, skeletal complex shape of vibrations in 1305–1000 cm^−1^ region of spectra (Figure 6). The spectral lines attributed to vibrations of dicyclopentadiene part of macromolecule chain are observed at 3040, 1613, 1272, 948 and 925 cm^−1^. The lines in 1700–1750 cm^−1^ region are attributed to oxidized macromolecules of the rubber under environmental conditions (light, oxygen and moisture) at storing after synthesis [29]. 

These lines are also observed in spectra of PIII treated rubber after PIII, except lines at 3040, 1600, 1270, 943 and 917 cm^−1^ attributed to vibrations of the dicyclopentadiene part of macromolecule (Figure 6). The intensity of these lines decreases in the spectra of PIII treated samples and these lines disappear at high fluence of treatment. Additionally, the wide band in 1750–1600 cm^−1^ region is observed in the spectra of PIII treated rubber. This band contains a number of overlapped lines, which are attributed to ν(C=C) and ν(C=O) vibrations of diene and aromatic structures and carbonyl, carboxyl and aldehyde groups in oxidized and carbonized surface layer of modified EPDM rubber. A contribution of C=N vibrations can be expected too. The new lines observed at 1598 and 1498 cm^−1^ are attributed to vibrations of aromatic rings appeared in surface layer of the rubber. In a region of wagging γ(C-H) vibrations, three lines at 967 cm^−1^ (vinylene group), 909 cm^−1^ (vinyl group) and 887 cm^−1^ (vinylidene group) are observed. These lines correspond to vibrations in unsaturated hydrocarbon groups appeared after PIII treatment. 

The intensity of these new lines is lower than the basic vibration lines of EPDM macromolecules. It corresponds to the thin modified layer of the rubber (about 150 nm) in comparison with penetration depth (400–800 nm) of infrared light from Ge ATR crystal into rubber sample at spectra recording. With PIII fluence increase, the intensity of all new lines increases corresponding to the concentration of such groups is growing with increase of number of bombarding ions. 

For the quantitative analysis of rubber structure by spectra were normalized on the intensity of 2960 and 1462 cm^−1^ lines related to vibrations of EPDM rubber macromolecules as the internal standard. Such normalization is based on assumption, that the modified layer thickness is much smaller than the depth penetration of infrared beam in ATR effect at spectra recording. 

**Figure 7:**
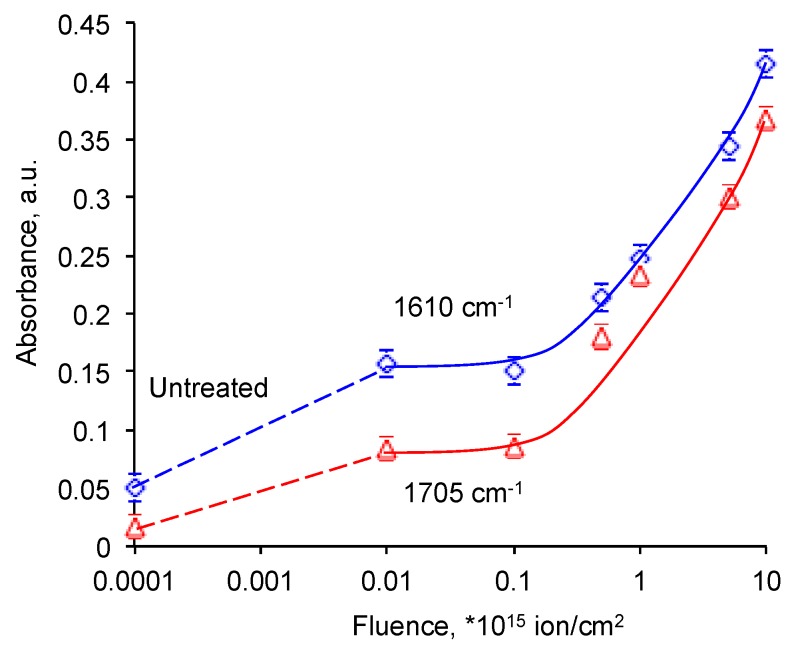
Normalized absorbance of ν(C=C) = 1610 cm^−1^ line and ν(C=C) = 1610 cm^−1^ line in FTIR ATR spectra of EPDM rubber with fluence of PIII.

The normalized intensities of C=O and C=C groups vibration lines are presented on Figure 7. The intensity of these lines grows with the PIII fluence. In the logarithm scale the increase is viewed sharp, while in the linear scale the curves have saturation. The logarithm scale is used for a good view of the all points. The increase corresponds to an appearance of new unsaturated and oxygen-containing groups in the surface layer of EPDM after PIII. The same increase was observed for other polymers after PIII [30]. 

The intensity of these group lines in the spectra of PIII treated EPDM at saturation (0.4) is significantly higher than the spectra of the plasma treated EPDM rubber (0.12) [28]. Even with periodical treatment the saturation level for plasma treated rubber is lower (0.19). The observed difference can be explained with deeper modified layer of EPDM rubber treated by PIII. The energy of ions in PIII is 20 kV that gives 100–150 nm thickness of the modified layer. The average energy of ions in plasma treatment is in a range of 0.01–0.1 eV that gives significantly lower thickness. However, a comparison of the depth based on an average ion energy in plasma is not completely correct due to a wide distribution of the ion energy in plasma discharge. Probably, the high energy tail of the energy distribution plays much significant role in the depth of the modified layer in plasma. 

**Figure 8:**
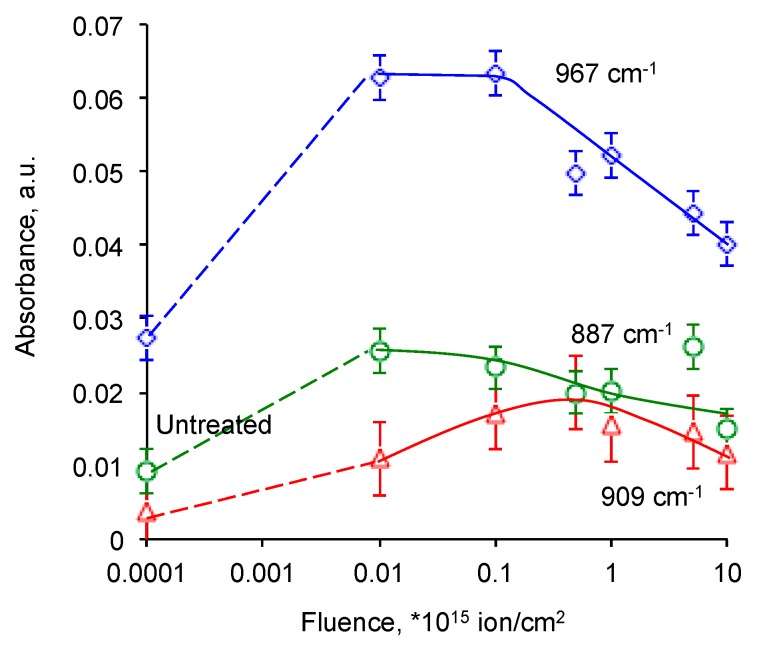
Normalized absorbance at 967 cm^−1^ line of vinylene group, at 887 cm^−1^ line of vinyliden group and at 909 cm^−1^ line of vinyl group in FTIR ATR spectra of EPDM rubber with fluence of PIII.

The intensity of 967 cm^−1^ line of vinylene group, 887 cm^−1^ line of vinyliden group and 909 cm^−1^ line of vinyl group increased with lower fluence of the PIII treatment (Figure 8). Then the intensity of 967 cm^−1^ line of vinylene group and 887 cm^−1^ line of vinyliden group goes down with the lowest values at the 10^16^ ions/cm^2^ fluence. The intensity of 909 cm^−1^ line of vinyl group grows up to maximum value at 10^15^ ions/cm^2^ fluence and then goes down with the lowest value at 10^16^ ions/cm^2^ fluence. 

**Figure A6:**
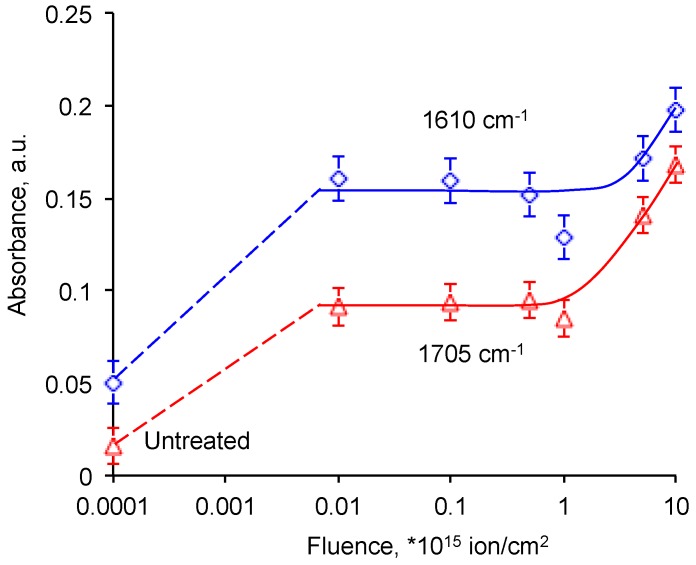
Normalized absorbance of ν(C=C) = 1610 cm^−1^ line and ν(C=C) = 1610 cm^−1^ line in FTIR ATR spectra of EPDM rubber with fluence of PIII without grid.

**Figure A7:**
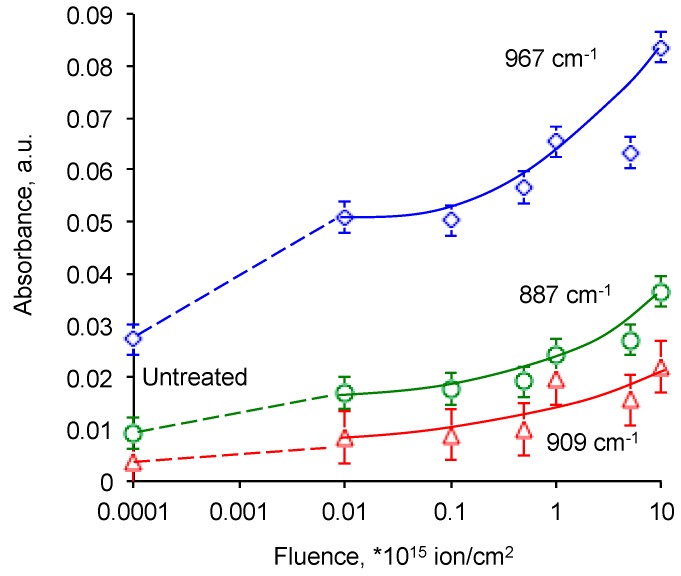
Normalized absorbance at 967 cm^−1^ line of vinylene group, at 887 cm^−1^ line of vinyliden group and at 909 cm^−1^ line of vinyl group in FTIR ATR spectra of EPDM rubber with fluence of PIII without grid.

The PIII treatment without the grid increases the intensity of of C=O and C=C groups vibration lines significantly smaller than with the grid (Figure A6 and Figure A7). The intensity of 967 cm^−1^ line of vinylene group, 887 cm^−1^ line of vinyliden group and 909 cm^−1^ line of vinyl group increases gradually with the PIII fluence, as it was observed for PIII treatment of polyethylene without the grid [39]. These results are consistent with an explanation of the electrical charge on the isolator surface at PIII treatment [30]. 

**Figure 9:**
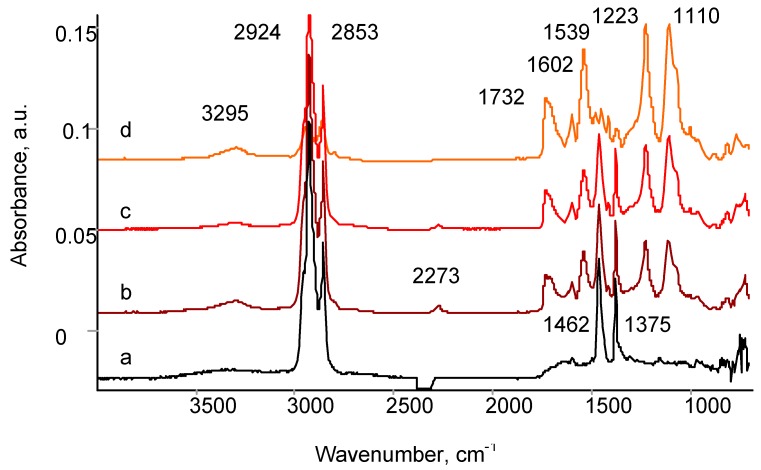
FTIR ATR spectra of peeled of adhesion joint: (**a**) initial PIII treated EPDM rubber; (**b**) PIII treated EPDM rubber peeled off from PU; (**c**) PU peeled off from PIII treated EPDM rubber; (**d**) initial PU surface.

The EPDM surface treated by PIII becomes active to polyurethane binder. The spectra of adhesion joint fracture surface for untreated EPDM rubber published before [40,41] showed an adhesive character of the fracture. The spectra of adhesion joint fracture surface for PIII treated EPDM rubber is presented in Figure 9. The spectrum of the surface fracture from adhesive site contains the lines of polyurethane adhesive at 3295 cm^−1^ (Amid A), 2942 cm^−1^ and 2857 cm^−1^ (stretching vibrations in CH_2_ group), 2273 cm^−1^ (residual NCO group), 1732 cm^−1^ (Amid I), 1602 cm^−1^ (aromatic ring), 1539 cm^−1^ (Amide II) and some lines of the EPDM rubber at 1462 cm^−1^ and 1372 cm^−1^ (bending vibrations in CH_2_ and CH_3_ groups). The spectrum of the surface fracture from EPDM side shows a similar spectra with higher intensity of EPDM lines and additional lines in the 1750–1600 cm^−1^ region attributed to ν(C=C) and ν(C=O) vibrations of the PIII modified layer. Therefore, the spectra of the fracture surface show that the fracture of the adhesion joint at the presence of solvent is cohesive in polyurethane binder and in EPDM rubber. The adhesive character of the fracture is not observed. 

## 5. Discussion

An adhesion of thermo-resistant rubber coating with the propellant is one of the key problems of solid fuel engine. A strong adhesion must be provided at a presence of high concentration plasticizer, in wide temperature range and high mechanical stresses during the engine operation. A failure of the adhesion can be a fatal event for the engine and for whole mission as it was observed in Space Shuttle Challenger disaster. 

Improving the adhesion in rubber-like materials to a polymer adhesive can be achieved by a high surface energy and wettability, developed roughness and chemical crosslinking of the polymer networks of rubber and adhesive. The last factor is critical for a good adhesion of polymer joints at the presence of high concentration of plasticizer, which can destroy all kinds of intermolecular interactions between macromolecules, except the chemical bonds. The achievement of the chemical crosslinking in the interface between two materials needs an activation of the rubber surface to specific groups of the adhesive during curing of the adhesive. However, the inertness of the rubber materials usually is associated with other technological properties. The example is EPDM rubber which thermo-resistant properties in combination with density, stability and mechanical strength make this candidate is one of the best materials for thermo-resistant coating. However, the EPDM rubber is a very inert material to all known adhesives of a polycondensation reaction of curing. The sufficient adhesion of the EPDM rubber requires a surface modification in such a way, that the surface layer of EPDM rubber could provide reaction centers for the same polycondensation reaction of curing. 

An effect of ion implantation into a polymer causes a deep structure transformations based on high energy processes at high density of defects in treated material. The high energy ion flies into polymer, collides electrons and atoms of macromolecules and transfers them the energy much higher than the covalent bond. The collided atoms fly deeper into the polymer, collide other electrons and atoms of other macromolecules and stopped when the kinetics energy is completely transferred and dissipated. As the result, the surface layer of polymer has a large number of disjoined atoms and electrons embedded into the polymer structure. 

The PIII treatment under investigated conditions causes the significant transformation of the EPDM surface layer structure in depth of 100–150 nm. The EPDM rubber contains only carbon and hydrogen atoms in the macromolecules. The hydrogen atoms have high volatility and likely are released from the surface layer as hydrogen gas. The surface becomes rich with carbon atoms. The carbon atoms with dangled bonds form new carbon structures. Following minimization of energy, the carbon structures, such as graphite plane and fullerene spheres are expected mostly to form in a short time after the ion penetration. Such carbon structures are observed in all carbon-base polymers. The appearance of the carbonized structures is observed in EPDM after PIII treatment. 

The graphitic-like structure is characterized by a presence of π-electron clouds. Aromatic-like structures are an excellent trapper of unpaired electrons, which can be stabilized for a long time in the structure due to delocalization effect. Such unpaired electrons belonging to the carbon atoms called free radicals are active to find a pair from any molecule with hydrogen or a weak chemical bond like epoxy ring, diene group or similar. The effect of chemical activity is observed by oxidation of the EPDM surface after PIII treatment. The free radicals have very universal character of the chemical activity. A combination of prolonged activity of the free radicals on p-electron clouds with high chemical activity makes them very efficient to bond any organic molecule from adhesive. 

The carbonized layer on PIII treated EPDM rubber provided sufficient activity of the EPDM surface to polyurethane with formation of covalent bonds between polymer networks. As a result, the mixture fraction of the adhesion joint is observed under high concentration of the solvent. Such a PIII treatment is a prospective method to provide the sufficient adhesion of the EPDM rubber to a polymer binder. 

## 6. Conclusions

The EPDM rubber was treated by nitrogen ions of 20 keV energy. The surface topology becomes developed with folds and cracks. The surface layer becomes carbonized and oxidized. The adhesion interface of EPDM rubber with polyurethane binder was improved up to the point where a covalent bond network was formed between the EPDM rubber and the polyurethane binder.
